# Allocation Mechanisms of the European Asylum, Migration and Integration Fund in Germany Raises Concerns

**DOI:** 10.1016/j.lanepe.2026.101741

**Published:** 2026-06-11

**Authors:** Andreas W. Gold, Kayvan Bozorgmehr

**Affiliations:** a Department Population Medicine and Health Services Research (AG 2), School of Public Health, Bielefeld University, Universitätsstraße 25, Bielefeld, Germany; b Section Health Equity Studies & Migration, Heidelberg University Hospital, Heidelberg University, Im Neuenheimer Feld 130.3, Heidelberg, Germany

As noted in this journal's May 2026 editorial, migration control has entered the clinic, thereby crossing a public health red line.^1^ We here argue that it has also entered the funding landscape, as the revised German programme for the European Asylum, Migration and Integration Fund (AMIF) reveals. These dynamics emerge against the backdrop of a shifting political landscape, in which migration policy is moving away from managing to mitigating, and from ordered to contained migration.

AMIF is a core EU instrument for reception and integration, with €10·94 billion allocated for 2021–27.^2^ A small portion of these funds is distributed at EU level, whilst the majority is allocated to the Member States, which administer them and can set their own priorities. Recipients of funding include private or public legal entities and non-governmental organisations. In Germany, AMIF is administered by the Federal Office for Migration and Refugees on behalf of the Federal Ministry of the Interior.^3^ Germanys revised national programme raises multiple concerns.

First, it expands funding opportunities for return counselling and infrastructural measures, while limiting support for people with pending asylum claims largely to ‘orientation courses’. This is a show case for the politicisation of migration and health, and may contribute to shrinking space for civil society and academia.^4^ It further creates considerable funding gaps for psychosocial counselling services and other support measures, which have the potential to directly impact the health and wellbeing of refugees.^5^

Second, the allocation mechanism raises further concerns. Although applications for funding could be submitted at any time between mid-2022 and early 2025, the process was suspended entirely between February and December 2025. The revised call for proposals was published in mid-December 2025 and was scheduled to remain open until mid-April 2026, with funding allocated on a *first-come, first-served* basis. However, applications for funding to support the integration of refugees exceeded the budget of €93·15 million by 30% just 27 hours after the call opened. Further applications were rejected due to budgetary constraints.

The *first come, first served* principle thus rewards speed over quality. It may further exacerbate the reduced availability of evidence-informed health and psychosocial interventions. Where full assessment of all applications is not feasible, fairer alternatives exist. A minimum open period and a lottery-based approach among eligible applications create equal opportunities and comparable conditions.^6^ Quality-based, transparent allocation processes would better align AMIF spending with health needs of refugees and foster equity in funding allocation.

## Contributors

AWG wrote the original and revised draft of the manuscript. KB reviewed and edited the manuscript. All authors reviewed and approved the final version of the manuscript and agreed to submit it for publication.

## AI- use statement

We used OpenAI's ChatGPT 5.2 and DeepL Write in order to refine language. After using these tools, the authors reviewed and edited the content as needed and take full responsibility.

## Declaration of interests

No funding was received for writing this manuscript. KB declares co-funding by the Asylum, Migration and Integration Fund (AMIF) of the European Union for the R&D project PROTECT.Ing (Ref.: 9168-2022-0104; 01. April 2023–30. September 2026) as grant holder. AWG declares that his position as a project coordinator and researcher at Heidelberg University Hospital is partially funded by the aforementioned R&D project PROTECT.Ing.

## References

[1] The Lancet Regional Health – Europe (2026). Migration control enters the clinic: a public health red line. Lancet Reg Health Eur.

[2] European Commission Asylum, Migration and Integration Fund (2021-2027). https://home-affairs.ec.europa.eu/funding/asylum-migration-and-integration-funds/asylum-migration-and-integration-fund-2021-2027_en.

[3] Federal Office for Migration and Refugees Asylum, Migration and Integration Fund 2021-2027. https://www.eu-migrationsfonds.de/SharedDocs/Anlagen/DE/AMIF/flyer-amif-en.pdf.

[4] Diaz E., Bojorquez I., Jouhaud R., Fabreau G., Blanchet K. (2026). Impact of a changing political environment on research on migration and health. Lancet Reg Health Eur.

[5] Bundesweite Arbeitsgemeinschaft der Psychosozialen Zentren für Flüchtlinge und Folteropfer e. V (2025). https://www.baff-zentren.org/wp-content/uploads/2025/10/Policy-Paper_Finanzierungsluecken-PSZ-1.pdf.

[6] Luebber F., Krach S., Martinez Mateo M. (2023). Rethink funding by putting the lottery first. Nat Hum Behav.

